# Procyclidine Use with Long-Acting Injectable Antipsychotics

**DOI:** 10.1192/bjo.2025.10367

**Published:** 2025-06-20

**Authors:** Prabin Gautam, Titilola Osoba, Shalina Mitchell

**Affiliations:** Kent and Medway NHS Trust, Dartford, United Kingdom

## Abstract

**Aims:** Our aim was to review if procyclidine is being prescribed as per BNF guidelines at DGS CMHT. As per BNF guidelines, procyclidine is recommended to be initiated at 2.5 mg of procyclidine three times per day increasing by 2.5 mg daily until symptoms are relieved. The effective maintenance dose is usually 10–30 mg procyclidine per day. After a period of 3–4 months of therapy, procyclidine should be withdrawn and the patient should be observed to see whether the neuroleptic-induced extrapyramidal symptoms recur.

**Methods:** A retrospective clinical audit was conducted on 36 patients receiving long-acting injectable antipsychotics at the Dartford, Gravesham, and Swanley Community Mental Health Team (DGS CMHT) between September 15, 2023, and January 7, 2024. Data was collected on patient demographics, diagnosis, antipsychotic medication, procyclidine use, Glasgow Antipsychotic Side-effect Scale (GASS) scores, and procyclidine review.

**Results:** The majority of patients were male (27 out of 36 [75%]) and in the 55–64 age range (16 out of 36 [44%]). The primary diagnoses were schizophrenia (25 out of 36 [69%]) and bipolar disorder (9 out of 36 [25%]). 14 out of 36 patients (39%) were currently taking regular procyclidine, with doses ranging from 5 mg once daily to 10 mg three times daily, while 6 were taking procyclidine as PRN. Regular procyclidine reviews were undertaken in 13 patients (92.9%), with review intervals ranging from monthly to 6-monthly. The common outcomes of reviews included dose adjustments, side effect monitoring, and discontinued use due to adverse effects or lack of efficacy. Out of those on regular procyclidine, 9 patients (64%) showed an improvement in their GASS scores. Among those on regular procyclidine, the starting dose was not available for 6 patients because the starting time pre-dates electronic records. From those included in our electronic records, the data indicates that the starting dose of procyclidine varied, with some patients being started on 5 mg as per need and later changed to regular, while others being started on 5 mg once a day, but none was started as per the trust recommended dosage of 2.5 mg three times a day. While there is no specific mention of a plan to review within 3–4 months for response to start of, or change in dosage of procyclidine, the data suggests, however, that regular reviews were being conducted to monitor the effectiveness and side effects of procyclidine. However, 4 patients, when they were first started on procyclidine, were asked to be reviewed by the GP.

**Conclusion:** The clinical audit demonstrates that procyclidine was being used to manage extrapyramidal side effects in patients receiving long-acting injectable antipsychotics at the DGS CMHT. The starting doses and review intervals for procyclidine varied, but regular monitoring of GASS scores and patient outcomes was occurring. The data suggests that procyclidine was generally effective in improving GASS scores and managing extrapyramidal symptoms, with 64% of patients showing improvement. It was worth noting that none of the patients in the record were started on the recommended starting dose of 2.5 mg TDS. Increasing awareness of trust protocol regarding prescribing of procyclidine is recommended to ensure evidence-based practice. This was presented in the local audit conference with team of doctors and pharmacists and changes implemented.

